# Analysis of 272 Genetic Variants in the Upgraded Interactive FXI Web Database Reveals New Insights into FXI Deficiency

**DOI:** 10.1055/a-1683-8605

**Published:** 2021-11-01

**Authors:** Victoria A. Harris, Weining Lin, Stephen J. Perkins

**Affiliations:** 1 Research Department of Structural and Molecular Biology, University College London, London, United Kingdom

**Keywords:** coagulation factors, haemostasis, protein structure/folding, inherited coagulation disorders, gene mutations

## Abstract

Coagulation Factor XI (FXI) is a plasma glycoprotein composed of four apple (Ap) domains and a serine protease (SP) domain. FXI circulates as a dimer and activates Factor IX (FIX), promoting thrombin production and preventing excess blood loss. Genetic variants that degrade FXI structure and function often lead to bleeding diatheses, commonly termed FXI deficiency. The first interactive FXI variant database underwent initial development in 2003 at https://www.factorxi.org . Here, based on a much improved FXI crystal structure, the upgraded FXI database contains information regarding 272 FXI variants (including 154 missense variants) found in 657 patients, this being a significant increase from the 183 variants identified in the 2009 update. Type I variants involve the simultaneous reduction of FXI coagulant activity (FXI:C) and FXI antigen levels (FXI:Ag), whereas Type II variants result in decreased FXI:C yet normal FXI:Ag. The database updates now highlight the predominance of Type I variants in FXI. Analysis in terms of a consensus Ap domain revealed the near-uniform distribution of 81 missense variants across the Ap domains. A further 66 missense variants were identified in the SP domain, showing that all regions of the FXI protein were important for function. The variants clarified the critical importance of changes in surface solvent accessibility, as well as those of cysteine residues and the dimer interface. Guidelines are provided below for clinicians who wish to use the database for diagnostic purposes. In conclusion, the updated database provides an easy-to-use web resource on FXI deficiency for clinicians.

## Introduction

**Fig. 1:**
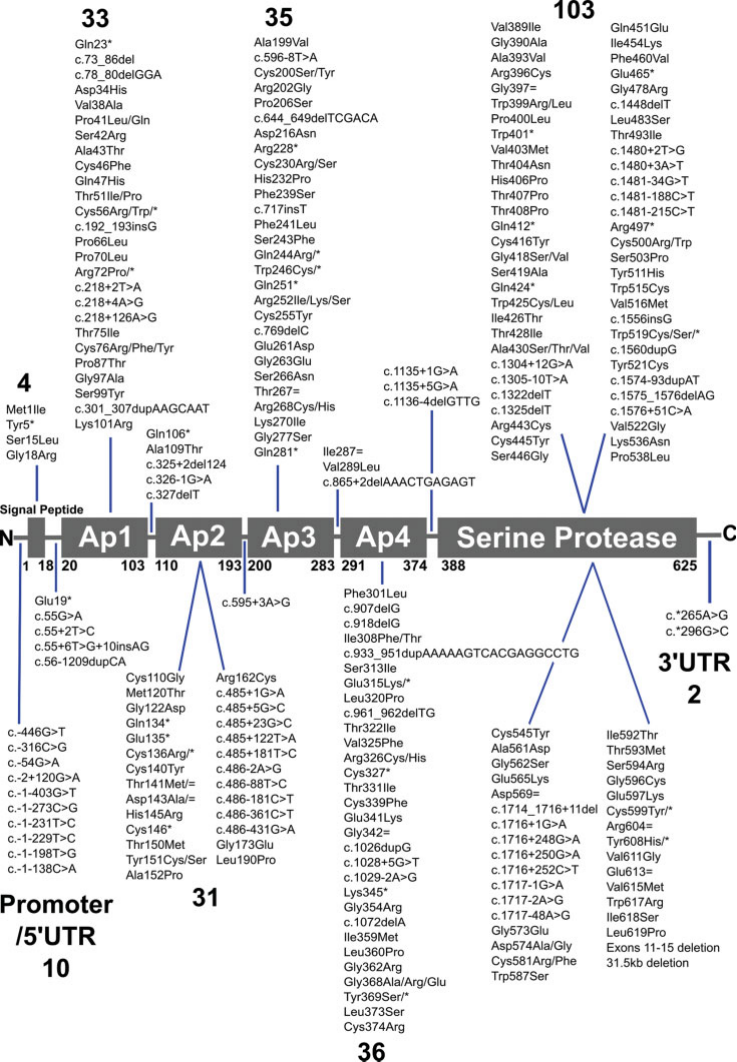
Distribution of the 272 variants identified within the *F11* gene. The Ap1-Ap4 and serine protease domains are drawn to scale. The number of variants in each of the respective domains and UTR regions is shown in large font above or below the variant lists. Intronic variants are included in their respective domains according to sequence numbering. The residue numbering in HGVS format (starting with 1 at the signal peptide) denotes the amino acids that start and end each domain. N and C represent the N- and C-termini of FXI respectively. Note that the two variants in the 3′UTR do not follow HGVS numbering.

Factor XI (FXI), a coagulation serine protease, is encoded by the *F11* gene located on the long arm of human chromosome 4 (4q35). The 23 kb gene comprises 15 exons that translate into a signal peptide, four apple (Ap) domains (Ap1-Ap4) and the catalytic serine protease (SP) domain ( Fig. 1 ). 1 2 The Ap domains in FXI are structurally homologous to each other and to those in human prekallikrein (PK), a zymogen protease involved in the kallikrein-kinin-system (KKS). Together such Ap domains form part of the plasminogen-apple-nematode (PAN) domain superfamily. 3 Specifically, FXI appeared to arise from the duplication of the PK gene, *Klkb1,* making FXI and PK paralogs of each other. The four Ap domains in each of FXI and PK form disk-like structures that are comprised of an antiparallel β-sheet attached to an α-helix through disulphide bridges. 4 5 FXI is synthesized as a 607 amino acid zymogen, which circulates in plasma in a dimeric form prior to activation. The dimer comprises two identical 80 kDa subunits, which are held together through non-covalent interactions between the two single Ap4 domains. The dimer is further stabilized by a Cys339-Cys339 interchain disulphide bridge between the Ap4 domains. The non-covalent interactions that stabilize dimer formation include hydrophobic ones as well as two Glu305-Lys349 and Asp307-Arg363 salt bridges. The activation of each FXI subunit involves the cleavage of the Arg387-Ile388 bond and can be driven by Factor XII (FXIIa), thrombin or by FXIa itself in a process known as autoactivation. 1 5 6 Once activated, FXIa cleaves zymogenic Factor IX (FIX) into FIXa. Subsequently, FIXa feeds into the coagulation cascade to promote thrombin production, aiding fibrin assembly and preventing excess blood loss. 1 7

Owing to the importance of FXI in coagulation, genetic variants that disrupt the native FXI structure and function lead to bleeding diatheses. Such disorders are most commonly referred to as FXI deficiency, but have been termed Haemophilia C, Rosenthal syndrome or Plasma Thromboplastin Antecedent deficiency in the past. 8 9 FXI deficiency occurs at a frequency of one in a million in the general population with higher incidence amongst the Ashkenazi Jewish population (one in 450 individuals). 10 11 12 Recent genetic variation studies of *F11* showed that FXI deficiency was found to be 2–20 times more frequent that expected in different ethnic groups. 13 Specifically, the heterozygous and homozygous frequencies within the Ashkenazi population are believed to be 9% and 0.22% respectively. In 1953, Rosenthal identified the first case of FXI deficiency in a Jewish family in the USA. 8 Many subsequent cases were identified in individuals of similar descent, with the nonsense mutation Glu135* (legacy numbering Glu117*) and the missense variant Phe301Leu (legacy numbering Phe283Leu) emerging as the founding causative variants. The most prominent mutations associated with FXI deficiency in Jewish populations are classified as being one of Type I-IV. The above point mutations are classified as Type II (Glu135*) and Type III (Phe301Leu) and remain as some of the most common FXI variants found today. Type I and IV mutations occur more sporadically and interfere with standard pre-mRNA splicing. Type I is a substitution and Type IV a deletion, both within intron N. 10 11 14 15 Despite the prevalence of FXI deficiency amongst the Jewish population, founding FXI variants have also been identified in other populations. Gln106* (legacy Gln88*) is a founding variant in French Nantes families, Cys56Arg (legacy Gln38Arg) in French Basques families, and Cys146* (legacy Cys128*) in English families. 15 16 17 The original nomenclature refers to FXI variants as having a cross-reacting material negative (CRM ^-^ ) or cross-reacting material positive (CRM ^+^ ) phenotype. CRM ^-^ (presently known as Type I) variants result in the simultaneous reduction of FXI coagulant activity (FXI:C) and FXI antigen (FXI:Ag) levels, most likely a result of the degradation of mutant FXI protein within cells. CRM ^+^ (presently known as Type II) variants result in a reduction of the FXI:C level but do not impact the FXI:Ag level. Such variants are most likely to be dysfunctional variants that go undetected by normal cellular quality control systems. FXI:C levels typically range from 70–150 IU/dL in unaffected individuals, while moderately deficient individuals have FXI:C levels ranging between 15–70 IU/dL and severely deficient individuals have levels <15 IU/dL. 2

What has been unclear is the lack of correlation between FXI activity, FXI deficiency and disease severity. To understand this relationship better, we created the first interactive web database for the coagulation proteins ( https://www.factorxi.org/ ) in 2003 for users, first published in 2005, and updated in 2009 to report 183 FXI variants. 18 19 We are not aware of other current dedicated databases for FXI mutations in Google searches. Other genetic repositories include the Exome Aggregation Consortium resource the Leiden Open-source Variation database (LOVD; http://www.lovd.nl/3.0/home ), 20 the Expert Protein Analysis system (ExPASy; https://www.expasy.org/ ), 21 the ClinVar resource ( http://www.ncbi.nlm.nih.gov/clinvar/ ), 22 and the public release of the Human Gene Mutation Database (HGMD; http://www.hgmd.cf.ac.uk/ac/index.php ). 23 To date, over 18,000 visits have been recorded on our FXI Web site. This interactive database has been upgraded it to include other coagulation proteins such as FIX and others. 24 These interactive variant databases bring key advantages of easy-to-use search and genetic and structural analysis tools for clinicians and scientists. In the present study, we now update and upgrade our FXI database to include an increased total of 272 variants in the *F11* gene ( Fig. 1 ) and the improved crystal structure from 2019. 25 The increased number of known and novel FXI variants clarifies the molecular basis of FXI deficiency. In particular, by comparisons with other coagulation proteases, we correlate the predominance of Type I (CRM ^-^ ) mutations within FXI with changes in surface solvent accessibilities of the affected residues, and with the occurrence of variants in cysteine residues. By focusing on the amino acid accessibilities in the closely-packed domain structure of the FXI dimer, in comparison with the extended domain structures in several other coagulation proteases, we explain the notable imbalance between Type I and Type II variants in FXI. The availability for clinicians of the upgraded Web site and the interpretation of deleterious variants significantly clarifies the molecular basis of FXI deficiency.

## Methods

### Source of the FXI Database

The interactive FXI web database at https://www.factorxi.org currently holds 272 genetic alterations in *F11* that are associated with FXI deficiency. The database was created at University College London, the Web site copyright is retained by S. J. Perkins and University College London, and database copying is not permitted without explicit permission from the author. The FXI database was initially populated in 2003 starting from a non-interactive Web site of the *F11* gene with 65 variants, together with literature searches of PubMed at https://pubmed.ncbi.nlm.nih.gov/ . 18 Further *F11* genetic variants were obtained from 32 patient records at the Haemophilia Centre and Thrombosis Unit at the Royal Free Hospital in London, as well as additional literature searches, making a total of 183 variants in 2009. 19 For the current database, the literature cut-off date was April 2021, giving an overall total of 272 unique variants found in 657 patients. These data were compiled into a spreadsheet and used to update the existing FXI MySQL database, using phpMyAdmin software ( https://www.phpmyadmin.net/ ) as an intermediary platform to the MySQL database. As a quality control, the original literature sources used for the 2005 and 2009 projects were re-consulted to re-validate and correct the entries if required. If required for personal or private research use, a list of the variants and their associated fields can be downloaded from the Variants menu on our Web site.

### Analysis of FXI Variants

The interactive database records DNA changes in Human Genome Variation Society (HGVS) format, where +1 refers to the A of the ATG initiation codon, at the start of the 18-residue signal peptide. As the default, protein changes are recorded in HGVS format, with codon +1 referring to the ATG initiation codon ( Fig. 1 ). To enable comparison with older FXI publications, the facility to use legacy numbering was included in the database for protein changes on the web database, with codon +1 referring to the first codon Glu19 of the mature FXI protein.

The original full-length FXI zymogen crystal structure at 0.287 nm structural resolution (PDB ID: 2F83) representing Cys20 to Thr622 (HGVS numbering) was recently superseded by an improved FXI zymogen crystal structure at 0.260 nm resolution (PDB ID: 6I58). 25 26 The latter was used here as the three-dimensional protein structural model on which the FXI variant analyses were based. The FXI dimer was created from the 6I58 structure using the Proteins, Interfaces, Structures and Assemblies (PDBePISA) server ( https://www.ebi.ac.uk/pdbe/pisa/ ). 27 While all structural analyses were performed using the 6I58 model, 95 additional FXI structures have become available in the protein database (PDB) since 2009 and are listed in our updated database. Of these, 92 crystal structures correspond to truncated FXI structures with only the serine protease domain present, and three are full-length FXI proteins in complexes with ligands. These structures do not show the full FXI zymogen in its native conformation and thus were not used.

The 6I58 crystal structure of unliganded FXI was analyzed using the Definition of Secondary Structure of Proteins (DSSP) tool at https://www3.cmbi.umcn.nl/xssp/ to determine the secondary structure of each FXI residue. 28 29 DSSP was applied both to the intact 6I58 structure as well as to the separated 6I58 domains. By this, residues were individually assigned to one of eight secondary structure types that were found in the crystal structure, namely one of H (α-helix), B (β-bridge), E (extended β-strand), G (3 _10_ helix), I (π-helix), T (hydrogen-bonded turn), S (bend) or C (undefined coil region). In addition, DSSP was used to determine the relative surface accessibility of each residue in the FXI crystal structure in Å ^2^ . The accessibilities were converted into % accessibility by dividing the DSSP output by the theoretical solvent accessible surface area of the amino acid sidechain in question. 28 29 30 The results were simplified as follows. Percentage accessibilities of 0–9% were given the value 0, 10–19% the value 1, 20–29% the value 2, and so on. Residues with accessibilities of 0 or 1 were classified as buried and those with accessibilities of 2–9 were classified as solvent-exposed.

The Ap domain secondary structure was comprised of a five-stranded antiparallel β-sheet with the β-strand topology C-E-D-G-A, a two-stranded B-F β-sheet and a centrally located α-helix (A1). The seven β-strands were labeled alphabetically (A-G) in the order in which they occurred in the sequence. The α-helix A1 and 3 _10_ -helices G1-G3 were similarly labeled in sequential order. 18 The six conserved cysteine residues of the Ap domains were numbered from C1 to C6 in the order they occurred in each Ap sequence. The three disulphide bridges between the α-helix and β-strands were denoted C1-C6, C2-C5 and C3-C4 and stabilized the folded Ap structure. 31 The SP domain was comprised of β-strands A-O, α-helices A1-A2 and 3 _10_ -helices G1-G5. As detailed previously, the Ap2 structure was used to represent the consensus Ap domain, owing to its low average root mean square deviation relative to the other three Ap domains after superimposition using the online secondary structure alignment program SSAP at http://www.cathdb.info/cgi-bin/cath/SsapServer.pl . 19 , 32 The interactions at the FXI dimer interface and between the Ap1-Ap4 and SP domains also utilized the PDBePISA tool. 27

To enable the clinician to assess the disruptive effect of a genetic variant on the FXI protein structure, four distinct substitution analyses were performed within the database on the F11 missense variants. Importantly, these monitor the extent of damage to the protein structure. These analyses were Polymorphism Phenotyping v2 (PolyPhen-2) ( http://genetics.bwh.harvard.edu/pph2/ ), Sorting Intolerant From Tolerant (SIFT) ( https://sift.bii.a-star.edu.sg/www/SIFT_seq_submit2.html ), Protein Variation Effect Analyzer (PROVEAN) ( http://provean.jcvi.org/seq_submit.php ) and Grantham analysis, using the Grantham matrix proposed in 1974. 33 34 35 36 Both the PolyPhen-2 and SIFT algorithms give variants a score ranging from 0.0 to 1.0. When using the PolyPhen-2 algorithm, the closer to 1.0 the prediction score is, the more damaging the variant is likely to be in the FXI protein structure. Conversely, SIFT scores closer to 0.0 predict more damaging variants in FXI and those closer to 1.0 predict those that are more tolerated in the protein. The PROVEAN threshold used was -2.5, thus variants with scores below the threshold were considered deleterious and those above the threshold were neutral. Grantham analysis differs from the other three in that it is not sequence specific, and is purely based on the amino acids undergoing substitution. Grantham scores range from 0 (silent variants) to 215, with larger scores indicating more radical amino acid substitutions that are likely to be more damaging to FXI.

## Results

### Classification of FXI Variants and Polymorphisms in the Updated Interactive Web Database

The interactive FXI web database ( https://www.factorxi.org ) currently presents information regarding 272 genetic variants ( Fig. 1 ) from 657 patient records, this being an almost 50% increase of 89 variants compared with the 2009 update, and an increase of 207 variants from the initial 2005 publication. 18 19 The 89 newer variants were sourced from 34 new research articles, increasing the literature pool by 30% from that in 2009. As well as the increased number of rare variants, the database has also been updated in terms of its interactive features, to follow our FIX Web site ( https://www.factorix.org ), where a site map facilitates user navigation. 24 The home page features two movies of the dimeric FXI and monomeric FXI structures with its variants, facilitating a three-dimensional visualization of the variant distribution. Allelic frequencies (AF) are also provided for variants when possible using the data supplied by the genome aggregation database (gnomAD) version 2.1.1 at https://gnomad.broadinstitute.org/ . 37 The gnomAD v2.1.1 dataset spanned 125,748 exome sequences and 15,708 whole-genome sequences and 117 (43%) of the 272 identified FXI variants were found in this. The AF was used as an indication of the relative frequency of a given variant at a specific genetic locus. The AF cut-off was taken as 0.01, thus an AF > 0.01 indicated a commonly-occurring variant. Of the 117 variants available in the gnomAD dataset, only 9 had AF > 0.01, all of which corresponded to known polymorphisms. The remaining 108 variants had AF < 0.01, highlighting that most FXI variants are rare. Using a more stringent AF cut-off of 0.001, only four additional variants occurred more frequently, leaving 104 rare FXI variants within the gnomAD dataset. Additional database features include a multiple sequence alignment of human FXI with other FXI species, to help users understand the phylogenetic history of the *F11* gene and the extent of residue conservation in related sequences. It should be noted that Java applets need to be enabled within the browser to permit the mutation map, the multiple sequence alignment and other related features to be seen. An interactive FXI structure is presented onto which missense variants can be mapped and analyzed for clearer structural and functional analysis of their consequence. Lastly, additional FXI structures and literature references provide a more up to date knowledge of FXI research.

**Fig. 2:**
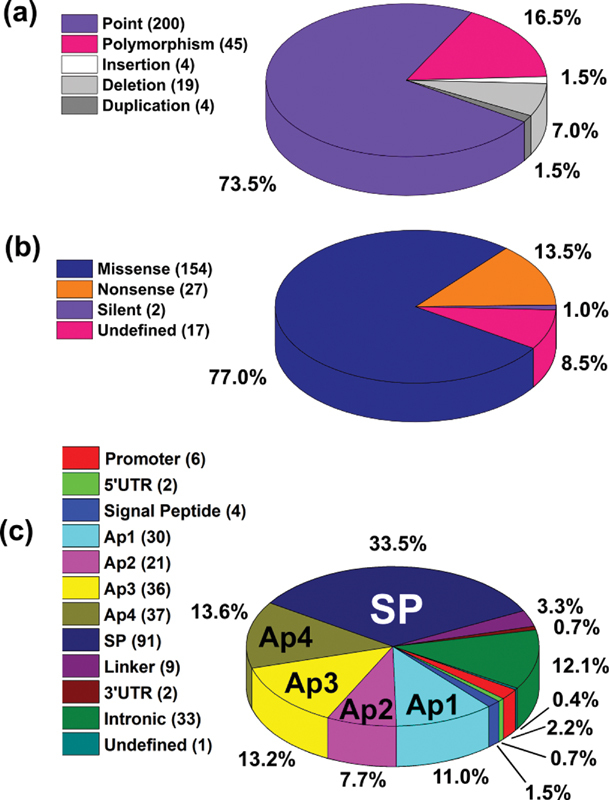
Distribution of the 272 variants found in the *F11* gene. The panels (a-c) indicate breakdowns of the 272 FXI variants into variant type, effect and location within the *F11* gene sequence. The charts illustrated here distinguish the non-disease associated polymorphisms from the disease-associated genetic variants. (a) The relative frequency of five different types of unique variants in the F11 gene. (b) Effect of the 200 point variants found in the F11 gene sequence. (c) Distribution of the 272 FXI variants across the *F11* gene sequence and FXI protein domains. Note, the variant in the undefined category is a 31.5 kb deletion that cannot be assigned to a single domain/region.

**Fig. 3:**
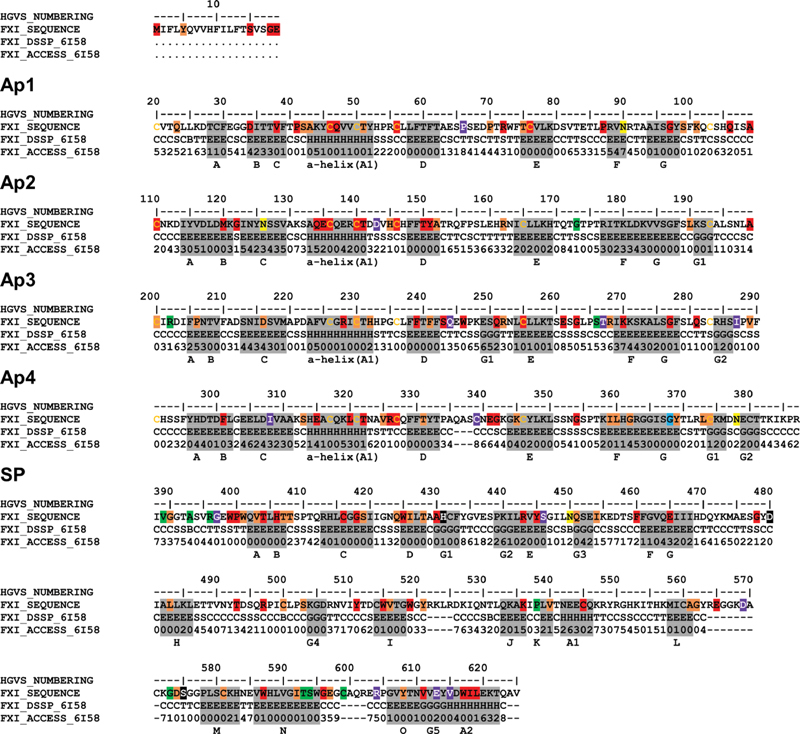
Secondary structure and accessibility analysis of variants in the FXI crystal structure. The FXI sequence is shown with the secondary structure assignments highlighted in gray boxes. Residues are denoted either H (α-helix), B (β-bridge), E (extended β-strand), G (3 _10_ helix), I (π-helix), T (hydrogen-bonded turn), S (bend) or C (undefined coil region). β-strands are labeled alphabetically in the order in which they occur. The β-strands in the Ap and SP domains are denoted A-G and A-O respectively. 3 _10_ -helices are denoted G1-G5 and α − helices are denoted A1-A2. The SP catalytic triad His431-Asp480-Ser575 residues (legacy numbering His413-Asp462-Ser557) are highlighted in black. The 24 conserved cysteine residues within the four Ap domains are shown in yellow text. Putative N-glycosylated residues are highlighted in yellow boxes. The positions of 198 point variants found in the *F11* gene are highlighted, where 75 red highlights denote Type I mutations, 11 green highlights denote Type II mutations, and 56 orange highlights denote mutations with unknown phenotype. The 13 purple highlights denote non-disease associated polymorphisms and the blue highlight denotes a Gly368 residue (legacy numbering Gly350) associated with both Type I and Type II phenotypes. Note that several highlights correspond to multiple variants at one residue position. All numbering is in HGVS format where the start of the signal peptide is denoted as Met1, rather than as Glu1 at the start of the mature protein.

Of the 272 variants identified in FXI, 227 are disease-associated (reported in research articles based on coagulation tests), and 45 are non-disease associated polymorphisms. The 272 variants can be classified by genetic event, of which point variants make up 73.53%, polymorphisms 16.54%, deletions 6.99%, duplications 1.47% and insertions 1.47% ( Fig. 2(a) ). The point variants can be further subdivided into missense (77.00%), nonsense (13.50%) and silent (1.00%) variants, with the remaining 8.50% of point variants being undefined ( Fig. 2(b) ). The variants are uniformly distributed throughout the FXI sequence, with variants found in all five domains and linker regions ( Fig. 2(c) ). Of the disease-associated variants, 96 (42.3%) are phenotypically classified as Type I, 12 (5.3%) as Type II and 119 (52.4%) as unknown. Here, Type II variants are characterized by a FXI:C to FXI:Ag ratio < 0.7. The Type I variants are scattered evenly across all domains whereas the Type II variants predominantly cluster in the SP domain (66.7%) ( Fig. 3 ). The two most common variants are Glu135* (legacy Glu117*) and Cys56Arg (legacy Cys38Arg), both of which are phenotypically classified as Type I. A total of 61 Glu135* and 36 Cys56Arg cases have been recorded in the web database. These are increases of five Glu135* and 24 Cys56Arg variants compared with the 2009 update. Other commonly occurring variants include Phe301Leu (legacy Phe283Leu), Cys146* (legacy Cys128*) and Gln281* (legacy Gln263*) of which there are 22 cases of each.

**Fig. 4:**
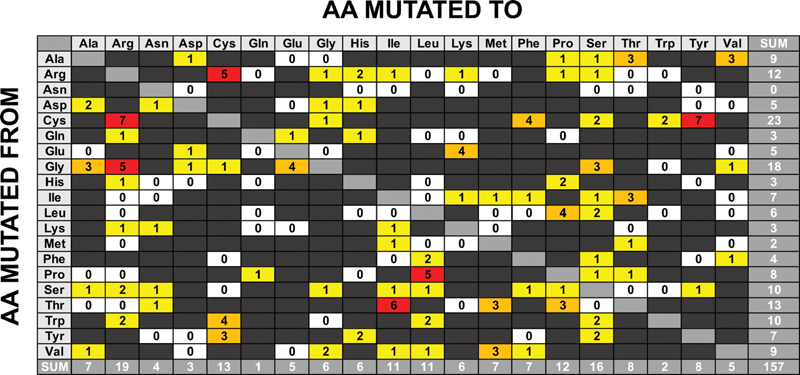
**
Substitution grid representing 157 missense variants (including eight polymorphisms) in the *F11* gene.** The grid illustrates the number of variants of each defined amino acid change. All substitutions shown are the result of a single nucleotide change. Any substitutions that require more than a single nucleotide change are shown in dark gray boxes. Silent variants are excluded from the grid and shown in pale gray. The white boxes represent possible substitutions that do not occur in the *F11* gene. The yellow boxes represent substitutions that occur once or twice, the orange boxes represent substitutions that occur three or four times and the red boxes represent substitutions that occur five or more times.

Substitution analysis was performed on 157 FXI missense variants to survey the distribution of the amino acid changes. The substitutions involved only single nucleotide changes; none appeared in the greyed boxes of Fig. 4 which would have signified two nucleotide changes. The total count for each missense change showed that Cys and Gly were the most commonly mutated residues (23 and 18 cases respectively), followed by others (right, Fig. 4 ). The predominance of Cys missense variants in the *F11* gene coincide with the predominance of Type I protein misfolding mutations in FXI, highlighting the importance of Cys residues in forming disulphide bridges in the protein structure to stabilize this. Gly residues with the smallest sidechain are the next most often mutated, this being explained by the detrimental effect of accommodating a larger sidechain in the FXI structure. The next four most often residue types to be mutated are the hydrophilic ones, namely Thr (13 cases), Arg (12 cases), and Ser/Trp (10 cases each). There is no clear rationale for these to be altered such that these become damaging to the protein, and this point is returned to below.

### Crystal structure analysis of secondary structures and accessibilities

The FXI protein structure rationalises the three-dimensional distribution of the variants. The previously used FXI zymogen crystal structure (PDB ID: 2F83) has now been superseded by a much improved one (PDB ID: 6I58). The 6I58 structure showed an improved structural resolution of 0.260 nm compared with that of 0.287 nm for 2F83 and this enabled better visualization and analysis of the distribution of variants in FXI. 25 26 When subjected to Ramachandran plot quality analysis ( http://www.ebi.ac.uk/pdbsum ), the 6I58 model gave an observed goodness-of-fit R-value of 0.216 when compared with the experimental X-ray data, compared with a larger R-value of 0.235 in the 2F83 crystal structure. In the 6I58 structure, 87% of amino acids were categorised in the “most favoured” conformational regions, 13% were in the “additional allowed” regions, and there were no conformational outliers. This outcome was improved compared with the 2F83 structure for which the corresponding figures were 74%, 20% and nine Ramachandran outliers respectively. 25 26 38 In Fig. 3 , 96 out of the 625 residues showed a different secondary structure assignment in the improved 6I58 structure compared with that in the 2F83 structure, even though the overall secondary structure was unaffected, and the changes affected mostly the loop conformations at the surface of the protein. From Fig. 3 likewise, 209 out of the 625 residues showed changes in surface accessibilities of at least 10% when comparing the 6I58 structure to 2F83, and 49 residues showed changes of more than 20%. The improved quality of the newer protein structure thus had clear effects on the analyses of the variants below.

**Fig. 5:**
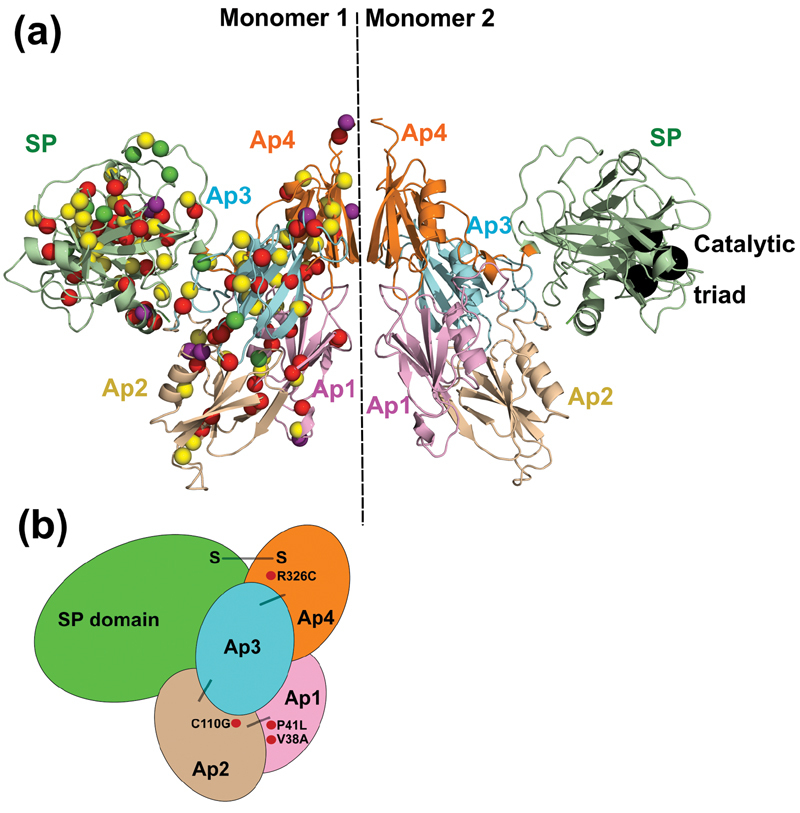
Structural and schematic view of variants within FXI. The dimer was created using its crystallographic symmetry, with the two Ap4 domains in contact with each other about an axis depicted as a dashed vertical line. (a) Distribution of 142 missense variants and 14 polymorphisms within dimeric FXI. The crystal structure of the FXI dimer is shown in ribbon format, with the Cα positions of variants and polymorphisms highlighted as spheres. Variant locations are illustrated in Monomer 1 and are colored according to phenotype. Type I (CRM-) variants are in red, Type II (CRM + ) variants in green, and those with unknown phenotype in yellow. The non-disease associated polymorphisms are depicted in purple. The catalytic triad His431-Asp480-Ser575 (legacy numbering His413-Asp462-Ser557) is shown in the SP domain of Monomer 2 as black spheres. The ribbon colors correspond to those used on our Web site https://www.factorxi.org . (b) Schematic representation of the five domains in monomeric FXI. The five domains are aligned and depicted in the same orientation and colors as in the ribbons in (a) above. The highlighted four variants, Val38Ala, Pro41Leu, Cys110Gly and Arg326Cys, are Type I variants found at interdomain contact points (see below).

The 6I58 structure was used to display 142 missense variants and 14 polymorphisms found in the FXI zymogen ( Fig. 5(a) ). The four Ap domains in each monomer were each composed of seven β-strands and an α-helix, which came together to form a five-stranded antiparallel β-sheet C-E-D-G-A, a two stranded B-F β-sheet and a central α-helix. This is most clearly seen for the Ap2 domain of Monomer 2 in Fig. 5(a) . Ap2, Ap3 and Ap4 also contained short C-terminal 3 _10_ -helices ( Fig. 3 ). The SP domain contained 15 β-strands, two α-helices and five short 3 _10_ -helices, all arranged as two subdomains, each of which flanked a substrate binding cleft between them ( Fig. 3 ). The catalytic triad of His431-Asp480-Ser575 (legacy His413-Asp462-Ser557) at the outer surface of the dimer is shown for Monomer 2 in Fig. 5(a) . In the full 6I58 structure, 322 residues out of 625 had percentage surface accessibilities of 0 or 1 (assigned as buried), 263 residues had accessibilities over 2 (assigned as surface exposed), and 40 residues were absent from the crystal structure and therefore not classified. The 6I58 structure revealed 98 variants with percentage surface accessibilities of 0 or 1, indicating sidechain burial, and 50 variants with accessibilities of 2 or more, indicating surface exposure ( Fig. 3 ). Note that the accessibilities of three variants in the SP domain were undeterminable due to structural limitations. Variant changes in buried positions are likely to interfere with intradomain interactions and overall protein conformation, accounting for the predominance of Type I variants in FXI, due to the high proportion of affected buried residues and the compact domain structure ( Fig. 5 ). In contrast, variants found at surface exposed locations are more likely to interfere with protein function without disturbing the overall structure. The low number of Type II variants in the Ap domains highlights their importance in maintaining the compact FXI structure, whereas the clustering of Type II variants in the catalytic SP domain indicates its significance in protein function rather than structure.

**Fig. 6:**
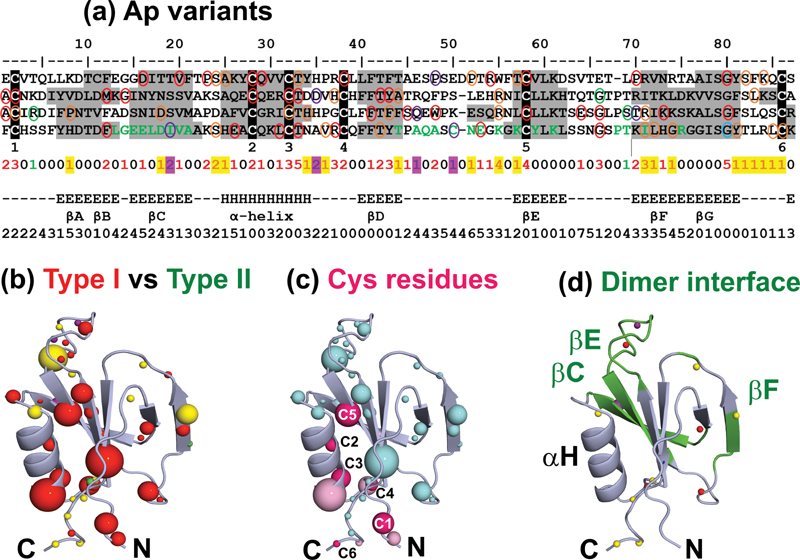
Mutational residue frequency in terms of a consensus Ap domain. A consensus Ap domain of length 87 residues shows the distribution of 90 Ap variants (missense and polymorphisms) in the four Ap1-Ap4 domains merged together. (a) The four Ap domain sequences are aligned to form a consensus, with the averaged secondary structure (Sec) and accessibility (Acc) of each residue listed directly below. The ‘Tot’ row illustrates the total number of variants found at each residue in the consensus. The six conserved Cys residues in each domain are highlighted in black and numbered 1–6 underneath the alignment. The Cys-Cys bridges occur at Ap1 (Cys20-Cys103, Cys46-Cys76, Cys50-Cys56), Ap2 (Cys110-Cys193, Cys136-Cys165, Cys140-Cys146), Ap3 (Cys200-Cys283, Cys226-Cys255, Cys230-Cys236) and Ap4 (Cys291-Cys374, Cys317-Cys346, Cys321-Cys327). Type I variants are circled in red, Type II in green and those of unknown phenotype in orange. Non-disease associated polymorphisms are circled in purple. The green residues in Ap4 mark those present at the FXI dimer interface. The ‘Sec’ rows highlight the α-helix region (H) and the five β-strand regions (E) in the consensus Ap domain. The β-strand regions are denoted AB, C, D, E and FG. The ‘Acc’ row denotes the relative accessibility of each consensus residue, where accessibilities of 0 or 1 indicate buried sidechains and accessibilities > 1 indicate sidechain exposure to solvent. (b) Variants are colored according to phenotype. Type I variants are colored in red, Type II in green and those of unknown phenotype in yellow. Polymorphisms are shown in purple. The sphere color represents the most commonly occurring phenotype at that position according to the sequence alignment in (a). The size of the spheres indicates the number of variants found at that position, and ranges from one to five. N and C refer to the N- and C-termini respectively. (c) Cys variants and their neighboring residues are shown as dark and light pink spheres respectively. All other variants are depicted in blue. The size of the spheres is again indicative of the number of variants found at that position. The six Cys residues are labeled C1-C6. ( **d** ) The FXI dimer interface is highlighted in green and the β-strands present at the interface are labeled. Variants found within Ap4 are shown as spheres, colored according to their phenotype as in (b).

A consensus Ap domain represents an average of the four Ap domains in FXI. The consensus enables all the Ap1-Ap4 variants to be shown in one view to determine features common to all four domains ( Fig. 6(a) ). The predominance of Type I variants (red) in the Ap domains is highlighted in Fig. 6(b) , where Type II variants (green) are almost absent. In this representation, Thr51 (legacy Thr33) (in a buried location at the end of the Ap α-helix) and Gly97 (legacy Gly79) (in a buried location at the end of the Ap β-sheet G) represent hotspots that disrupt the Ap domains.

### Analysis of Disulphide Bridges and Cys Variants in FXI

Further analyses focused on specific details of the FXI structure. The most commonly mutated residue in FXI are the Cys residues ( Fig. 4 ). The covalent links formed by 17 disulphide bridges in a FXI monomer are key to the structure and function of FXI. Each Ap domain possesses three intrachain disulphide bridges C1-C6, C2-C5 and C3-C4 (black highlights in Fig. 6(a) ), the first of which stabilizes the link between the N-terminus and C-terminus of each Ap domain. There is a free Cys29 residue in Ap1. The SP domain has five bridges (Cys380-Cys500, Cys416-Cys432, Cys514-Cys,581, Cys545-Cys560, Cys571-Cys599). The Ap4 domains form an additional interchain disulphide bridge at Cys339-Cys339 to stabilize dimer formation. 1 31 39 40 Type I Cys variants disrupt disulphide bridge formation, destabilizing the protein structure and leading to a FXI deficient state. A total of 28 distinct Cys variants have been identified within FXI, 20 of which are in the Ap domains and eight are in the SP domain. Of the 28 variants, 12 have Type I phenotypes (Cys46Phe, Cys56Arg/Trp, Cys76Tyr, Cys110Gly, Cys136*, Cys140Tyr, Cys146*, Cys255Tyr, Cys327*, Cys416Tyr, Cys545Tyr), one has a Type II phenotype (Cys599*) and one is a non-disease associated polymorphism (Cys339Phe). The remaining 14 variants do not have defined phenotypes (Cys56*, Cys76Arg/Phe, Cys136Arg, Cys200Ser/Tyr, Cys230Arg/Ser, Cys374Arg, Cys500Arg/Trp, Cys581Arg/Phe, Cys599Tyr) ( Fig. 3 ). The 13 substitution variants that introduce new Cys residues into FXI predominantly possess Type I phenotypes (Trp246Cys, Arg326Cys, Trp425Cys, Arg443Cys, Tyr445Cys, Trp515Cys, Trp519Cys, Gly596Cys), with only one possessing a Type II phenotype (Arg396Cys in the SP domain) and four with unknown phenotypes (Tyr151Cys, Arg162Cys, Arg268Cys, Tyr521Cys). All individuals with such variants in a homozygous or compound heterozygous form exhibit a severe FXI deficiency. In addition to the Cys residues being Type I mutational hotspots, variants that are adjacent to Cys residues are also able to perturb the disulphide bridge packing within the protein fold ( Fig. 6(c) ).

### Analysis of Variants at the FXI Dimer Interface

The PDBePISA tool was used to identify the 17 Ap4 contact residues involved in FXI dimerization with buried surface area changes of over 5 Å ^2^ (Leu302 to Val309, Thr333, Cys339, Asn340, Lys345, Tyr347, Lys349, Thr357, Leu360, Arg363) (green in Fig. 6(a) ). 22 Specifically, Cys339 forms an interchain disulphide bond, which is important for stabilization but is not essential for dimer formation or functionality. 39 There are 36 Ap4 variants in total, yet only three are found at the dimer interface (Ile308Phe/Thr, Cys339Phe), highlighting the importance of the conservation of residues at the dimer interface ( Figs. 6(a) and 6(d) ). Ile308Phe/Thr and Cys339Phe are non-disease associated polymorphisms. Given that Ile, Phe, Thr, and Cys have neutral side chains, the Ile308Phe/Thr and Cys339Phe substitutions are unlikely to have a large impact on the non-covalent dimerization interactions.

### Comparison of Variant Phenotypes with Damage Analyses and Residue Accessibilities

**Fig. 7:**
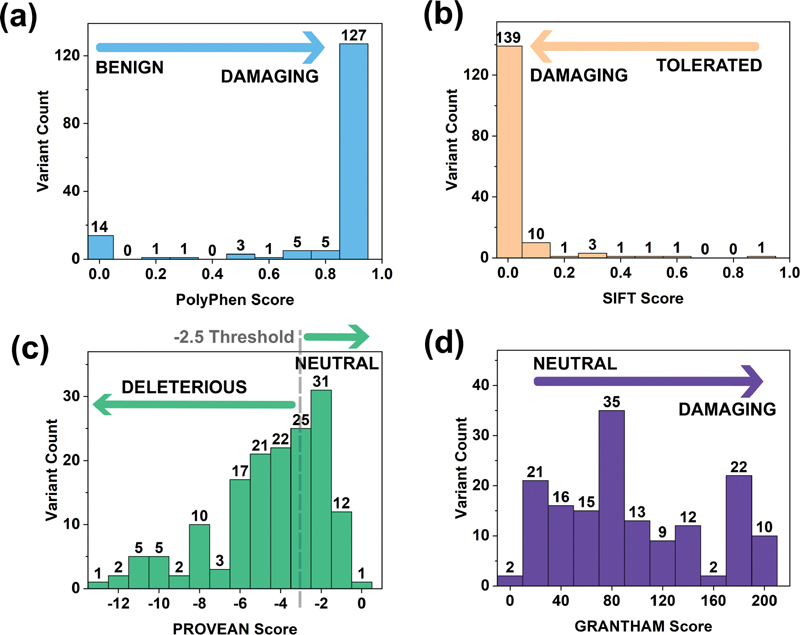
Substitution analysis of 157 missense variants (including eight polymorphisms) in the *F11* gene. Substitution analyses predict the damaging effects of substitution variants. (a) Graphical distribution of variants determined by their PolyPhen-2 score. (b) Graphical distribution of variants determined by their SIFT score. (c) Graphical distribution of variants determined by their PROVEAN score. The PROVEAN threshold used was -2.5. (d) Graphical distribution of variants determined by their Grantham score.

The observation of a missense FXI variant in the gene of a patient is not necessarily causative of a bleeding disorder. To assist clinical interpretations, the FXI Web site offered guidelines to clarify whether a given variant is likely to disturb the overall protein structure and function. The PolyPhen-2 algorithm predicted that 127 (80.9%) of the 157 missense variants to be damaging, with scores of 0.9 to 1.0 ( Fig. 7(a) ). Similarly, SIFT analysis predicted that 139 (88.5%) of the missense variants were damaging ( Fig. 7(b) ). Such findings indicate that most FXI variants are causative for FXI deficiency. The PROVEAN and Grantham analyses demonstrated less clear trends in which the predictions of the damaging scores fell across a larger scale with less clear outcomes ( Figs. 7(c,d) ). The interactive FXI database thus provided variant-specific PolyPhen-2, SIFT, PROVEAN and Grantham scores for the 157 missense variants, thus providing an easy-to-use clinical support tool to clarify the significance of a variant.

**Fig. 8:**
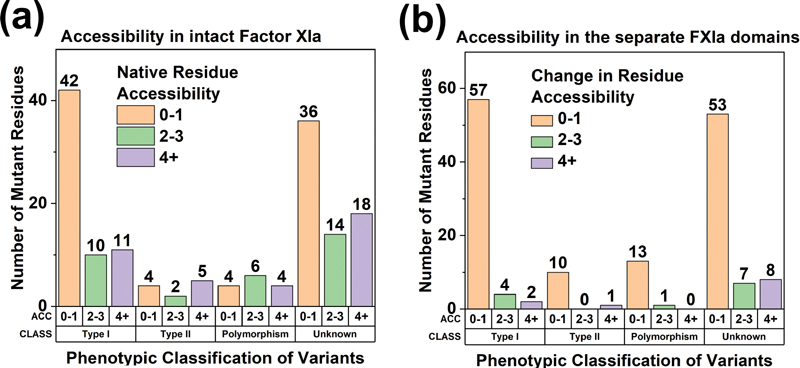
Accessibility analyses of 142 missense variants and 14 polymorphisms in the FXI protein structure. (a) FXI variants in the FXIa structure are grouped by phenotypic classification (CLASS). The variants are further subdivided according to the native residue accessibility (ACC) of the intact protein. Accessibility was determined using DSSP and is explained in detail in the Methods section. Accessibilities of 0 or 1 indicate sidechain burial and values >1 indicate sidechain exposure to solvent. (b) FXI variants are again grouped by phenotypic classification (CLASS) and accessibility (ACC). Here, accessibility refers to the change in residue accessibility when the intact FXI protein is separated into five distinct domains, namely the Apple1, Apple2, Apple3, Apple4 and SP domains.

To further examine the relationship between FXI phenotypes and surface accessibilities in the FXI protein structure, the residue surface accessibilities were displayed as a function of phenotype for FXI and the individually separated FXI domains. The high proportion of Type I variants was thus investigated by calculating the surface accessibilities of 142 FXI missense variants and 14 polymorphisms using the PDBePISA tool for the intact FXI protein and the individually separated FXI domains ( Fig. 8 ). Notably a high 66.7% of Type I variants (42 of 63) showed accessibilities of 0 or 1, highlighting their predisposition to be buried within the FXI protein structure ( Fig. 8(a) ). In contrast, Type II variants and polymorphisms appeared at both exposed and buried regions of the FXI structure, with no clear preference for either location. Many variant residues were of unknown phenotype, however interestingly the majority of these showed low accessibilities. In calculations made following domain separation, the resulting changes in accessibility compared with the intact protein enabled the identification of residues that made interdomain contacts. An even higher proportion of 90.5% of Type I variants (57 of 63) were located to residues that showed small accessibility changes after domain separation ( Fig. 8(b) ). The same outcome was also seen for Type II variants, polymorphisms and unassigned phenotypes. The predominance of Type I variants (and others) at such sites illustrates that small perturbations in the FXI structure, through the introduction of variants that lead to slight changes in surface accessibility, are sufficient to inactivate the protein and lead to disease states.

**Fig. 9:**
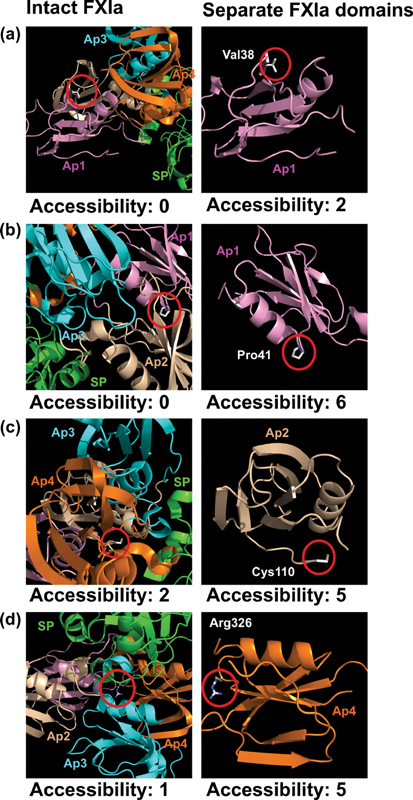
Molecular graphic representation of residues within the FXI protein structure. The left-hand panel highlights the residue of interest and its calculated solvent accessibility in the native FXI protein. The right-hand side panel shows the same residue in its respective isolated domain, with its corresponding accessibility. Panels (a), (b), (c) and (d) highlight the residues Val38, Pro41, Cys110 and Arg326 respectively. Accessibility calculations are detailed in the Methods section. The domain coloring corresponds to that in Figure 3 . All residue numbering is given in HGVS format.

For further insight into the above outcome, four individual variant residues were visually highlighted. The FXI Ap domains were tightly packed together to form a compact structure with intricate interdomain interactions. Four distinct residues associated with Type I variants were identified, namely (a) Val38Ala, (b) Pro41Leu, (c) Cys110Gly and (d) Arg326Cys ( Fig. 9 ). Following domain separation, the accessibilities of these four residues increased significantly, corresponding to a transition from burial to exposure. These accessibility changes indicated the extent to which the residues are packed together against surrounding domains. Missense variants at such locations will perturb these interdomain interactions, disrupting the native FXI structure and resulting in premature protein degradation. Thus, we have provided a molecular explanation for the relative abundance of Type I variants in FXI in terms of small but significant disruptions to the tightly packed domain structure.

## Discussion

The new datasets for FXI in this study have significantly improved the quality of the analyses of variants compared with our previous study, 19 and lead to further clinical insights into the occurrence of FXI disease states. Given the known inability to correlate FXI activity, FXI deficiency, and disease severity, it is unlikely that the updated FXI database will provide a clear answer to this conundrum. Some patients seem to need FXI, while others do not. Specific FXI variants have not been linked to more severe phenotypes. The cause of the variability in symptoms in FXI-deficient individuals may lie outside the FXI protein. Nonetheless the updated FXI database now provides clearer insights into the role played by the variants. This upgrade has benefitted from three main advances: (a) the availability of an additional 50% of reported rare variants from literature sources to make a total of 272 variants ( Fig. 1 ); (b) the significantly improved crystal structure for the FXI zymogen 25 ; (c) the upgrade of the previous FXI Web site user interface into that similar to the UCL coagulation Factor IX Web site. 24 The inclusion of the disruption scores from Polyphen-2, SIFT, PROVEAN and Grantham offers new insight into the impact of the variants and will provide the clinician with guidelines on the significance of a variant, based on the surveys of Fig. 8 . Most notably, we show that these variants are found across the FXI protein structure, and that accessibility changes in the packing arrangement of amino acid residues in the folded FXI structure by residue substitution is a major cause of FXI deficiency. Accessibility changes as well as the disruption scores may be a good predictor for the damaging changes associated with a variant.

In the coagulation proteases, FXI presents unique features by virtue of its compact protein domain structure. There are other proteins that likewise possess compact domain structures, such as the serine protease Factor I of the complement cascade of immune defense that contains five domains in contact with each other (complement proteins are evolutionarily related to coagulation proteins). Like FXI, Factor I shows that variants are distributed throughout the protein structures, implying that any of these will perturb the correctly folded protein structure. However, Factor I is monomeric and not dimeric as is FXI. 41 In contrast, three-dimensional structures for Factors VII, IX and X (FVII, FIX and FX) present extended domain arrangements based on the four domains termed Gla-EGF1-EGF2-SP. Where the phenotypes are known, FVII, FIX and FX variants show a higher proportion of Type II phenotypes and are associated with functional defects, rather than Type I. 19 42 This outcome is as expected given that these three proteins have extended domain arrangements. For proteins that are dominantly affected by functional defects, such as Factor H and C3 of complement, these show tendencies to reveal “hot-spots” where genetic variants accumulate in small but functionally important regions of the protein structure. Certain types of variants do not exist in FXI. There are no variants reported that affect the catalytic residues His431-Asp480-Ser575 that make up the peptide cleavage site in FXI. Likewise, there are no variants reported that prevent FXI from forming dimers. Very few of the contact residues at the dimer interface are associated with variants, and those that do only show minor perturbations to the protein structure.

Specific residue types are becoming more abundant as the number of observed genetic variants increase. In this study, Cys residues were flagged up as being a frequent source of disease-associated variants in FXI ( Fig. 6(c) ). Cys residues are important for the stability and functionality of FXI, and there are 18 disulphide bridges in a FXI monomer. Unsurprisingly the breakage of a Cys-Cys disulphide bridge is expected to impact severely on the FXI protein. The higher frequency of variants at Cys32 and Cys58 was already evident in the consensus Ap domain in 2009 where all six Cys residues were associated with variants. 19 In the present study, the involvement of all six Cys residues in variants in the consensus Ap domain was verified ( Fig. 6(a) ). A similar outcome was also recently noted with the consensus short complement regulator domain in the complement proteins, which possesses two conserved disulphide bridges. Initially the Cys residues were not prominent as variant hotspots, but the most recent update of the web database showed that these were prominent with 5–13 occurrences. 43 44

**Fig. 10:**
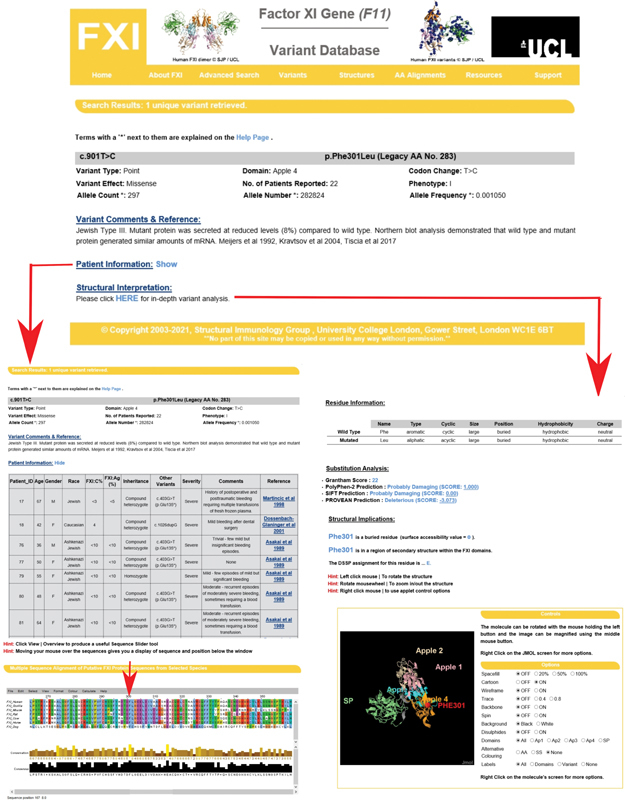
Screenshots of the upgraded FXI website to illustrate the analysis made for the Phe301Leu variant. The upper panel displays the output when residue 301 is inputted on the home page. By clicking “Show” on the patient information, the lower left panel lists genetic information for the 22 patients reported with Phe301Leu variant, of which the first five records are visible, together with the source of the patient record. The sequence alignment is shown underneath with Phe301 highlighted. Clicking “HERE” on the structural interpretation gives the image shown on the bottom right panel. This assesses the buried or exposed accessibility of the variant and its location in the FXI protein structure. A JMol view of the FXI structure is displayed that can be rotated and zoomed into as desired. The substitution analysis to predict the damaging effects of each missense variant (PolyPhen-2, SIFT, Grantham and PROVEAN) is provided to facilitate clinical diagnosis.

Our interactive FXI database will serve as a useful resource for clinicians and scientists to diagnose FXI deficiency and predict variant effects. Database technology becomes required in the light of the large increases in the known genetic variants in FXI, when a simple flat list became no longer adequate to monitor these. The Web site layout is designed to present genetic and structural information on FXI as two distinct but parallel themes, similar to that for our original FXI database 18 19 and the FIX database. 24 This is illustrated using genetic and structural outputs for the established Phe301Leu variant (the Jewish Type III variant with legacy numbering Phe283Leu), for which 22 patient records exist ( Fig. 10 ). On the left, further insight on the conservation of Phe301 is obtained from the AA Alignments tab which shows Phe301 aligned with six other mammalian species to show that this residue is fully conserved and therefore essential for FXI function. On the right, the structural analysis shows that Phe301 is a buried residue on a β-strand, and the JMol viewer shows that this is located inside the Apple 4 domain. Further research into FXI will be key to understanding the relationship between FXI deficiency and disease severity, including experimental studies. While the improved 2019 crystal structure for the FXI zymogen has greatly facilitated variant analysis, a crystal structure for activated FXIa will further help explain the molecular basis of FXI deficiency. There may be a large conformational difference in FXIa compared with FXI, by analogy with the structure of activated plasma kallikrein compared with the FXI zymogen; kallikrein is homologous to FXI. 25

## Notes

**What is Known on this Topic**

(1) Many more FXI variants have been published in the literature since we last updated our interactive web database at https://www.factorxi.org in 2005 and 2009.
(2) The crystal structure of the FXI zymogen dimer enables an assessment of the damaging effects of FXI missense variants, and these need updating in the light of the significantly improved FXI crystal structure from 2019.

**What this Paper Adds**

(1) Of the 272 unique variants from 657 case reports that are now in our interactive database from literature searches, 227 are disease-associated, the majority of which are phenotypically classified as Type I, and 45 are non-disease-associated.
(2) We explain the molecular basis of many Type I variants in FXI in terms of the altered surface accessibility of the affected residues, the importance of affected Cys residues in disulphide bridges, and perturbations of the Ap4-Ap4 contacts that form the FXI dimer.
(3) Our interactive FXI Web site was upgraded for improved ease of clinical use, to enable the better utility of sequences and structural modelling to analyze FXI genetic variants and assess their damaging effects.
